# Impact of a Loss-of-Function Variant in *HSD17B13* on Hepatic Decompensation and Mortality in Cirrhotic Patients

**DOI:** 10.3390/ijms231911840

**Published:** 2022-10-06

**Authors:** Antonio Gil-Gómez, Ángela Rojas, María R. García-Lozano, Rocío Muñoz-Hernández, Rocío Gallego-Durán, Douglas Maya-Miles, Rocío Montero-Vallejo, Sheila Gato, Javier Gallego, Rubén Francés, Germán Soriano, Javier Ampuero, Manuel Romero-Gómez

**Affiliations:** 1 SeLiver Group, Institute of Biomedicine of Seville (IBiS), Virgen del Rocio University Hospital, CSIC, University of Seville, 41013 Seville, Spain; 2 CIBERehd, Instituto de Salud Carlos III, 28029 Madrid, Spain; 3 Clinical Medicine Department and IDIBE, Miguel Hernández University, 03550 San Juan de Alicante, Spain; 4 IIS ISABIAL, Hospital General Universitario de Alicante, 03010 Alicante, Spain; 5 Department of Gastroenterology, Hospital de la Santa Creu i Sant Pau, 08025 Barcelona, Spain; 6 UCM Digestive Diseases, Virgen del Rocío University Hospital, 41013 Seville, Spain

**Keywords:** cirrhosis, polymorphism, *PNPLA3*, *HSD17B13*, NAFLD, fibrosis, ascites, hepatic encephalopathy, SNP, hepatic decompensation

## Abstract

A common splice variant in *HSD17B13* (rs72613567:TA) was recently found to be associated with a reduced risk of developing chronic liver disease in NAFLD patients and a reduced risk of progression to advanced fibrosis and cirrhosis. In this study, we aimed to evaluate the prognosis of cirrhotic patients harboring this variant. We performed a retrospective analysis on 483 prospectively recruited patients from four different hospitals in Spain, followed-up for at least 5 years. We collected clinical, demographic, and biochemical data, and we performed a genotyping analysis for common variants previously associated with liver disease risk (*HSD17B13* rs72613567:TA and *PNPLA3* rs738409). Patients homozygous for the TA allele showed a higher MELD score (*p* = 0.047), Child–Turcotte–Pugh score (*p* = 0.014), and INR levels (*p* = 0.046), as well as decreased albumin (*p* = 0.004) at baseline. After multivariate analysis, patients with the “protective” variant indeed had an increased risk of hepatic decompensation [aHR 2.37 (1.09–5.06); *p* = 0.029] and liver-related mortality [aHR 2.32 (1.20–4.46); *p* = 0.012]. Specifically, these patients had an increased risk of developing ascites (Log-R 11.6; *p* < 0.001), hepatic encephalopathy (Log-R 10.2; *p* < 0.01), and higher mortality (Log-R 14.1; *p* < 0.001) at 5 years of follow-up. Interactions with the etiology of the cirrhosis and with the variant rs738409 in *PNPLA3* are also described. These findings suggest that the variant rs72613567:TA in *HSD17B13* has no protective effect, but indeed increases the risk of decompensation and death in patients with advanced chronic liver disease.

## 1. Introduction

Chronic liver disease progresses from fibrosis to cirrhosis, and ultimately to hepatic failure and hepatocellular carcinoma (HCC), being prominent causes of illness and death globally. The most common etiologies of cirrhosis are alcohol consumption, chronic hepatitis C or B infection, and non-alcoholic fatty liver disease (NAFLD), whose prevalence is increasing in parallel with that of metabolic syndrome [1,2]. Cirrhosis progression results in portal hypertension and hepatic decompensation, both of which substantially increase the risk of mortality, accounting for approximately 2 million deaths worldwide [3]. Notably, liver disease progression shows considerable interindividual variability; it has been estimated that host genetic factors could explain approximately half of this variability, contributing to the progression of cirrhosis and predisposing patients to the development of liver-related events [4,5].

Genome-wide association studies have identified sequence variations with robust effects on multiple stages of the disorder. The most validated so far is the variant p.I148M in 1-acylglycerol-3-phosphate O-acyltransferase (*PNPLA3)*, associated with higher triglyceride levels, as well as with an increased risk of non-alcoholic steatohepatitis, cirrhosis decompensation, HCC development, and mortality [6,7,8,9]. Recently, an indel variant (T > TA variant, rs72613567) in the 17-beta-hydroxysteroid dehydrogenase 13 (*HSD17B13*) gene, leading to the production of a truncated protein with reduced activity, has been associated with decreased aminotransferase levels and a reduction of liver injury in the setting of a fatty liver, mitigating the risk conferred by the carriage of the *PNPLA3* variant [10,11]. Additionally, this variant has been associated with lower odds of developing cirrhosis and HCC among alcohol misusers [12,13]. However, the physiological role of this protein and the mechanisms underlying these associations have yet to be elucidated, since much of the genetic risk of chronic liver disease remains unexplained.

The goal of this study was to evaluate the clinical impact of the genetic variant rs72613567:TA in *HSD17B13* on the prognosis of cirrhotic patients and to explore possible risk interactions with the underlying etiology or the genotype of *PNPLA3* rs738409.

## 2. Results

### 2.1. Baseline Characteristics and Genotyping

The clinical and demographic characteristics of the patients are shown in Table 1. The majority of the patients were male (68.7%), with a mean age of 59.4 ± 11.9 years old. The most frequent etiology was alcohol (48.7%), followed by viral hepatitis (35.8% C and 6.2% B), with the other 9.3% being NAFLD, primary biliary cirrhosis, or autoimmune-related cirrhosis. At the time of inclusion, liver function was CTP score A, with a Model for End-stage Liver Disease (MELD) score of 12.2 ± 5.1, in 48.9% of patients.

**Figure 1:**
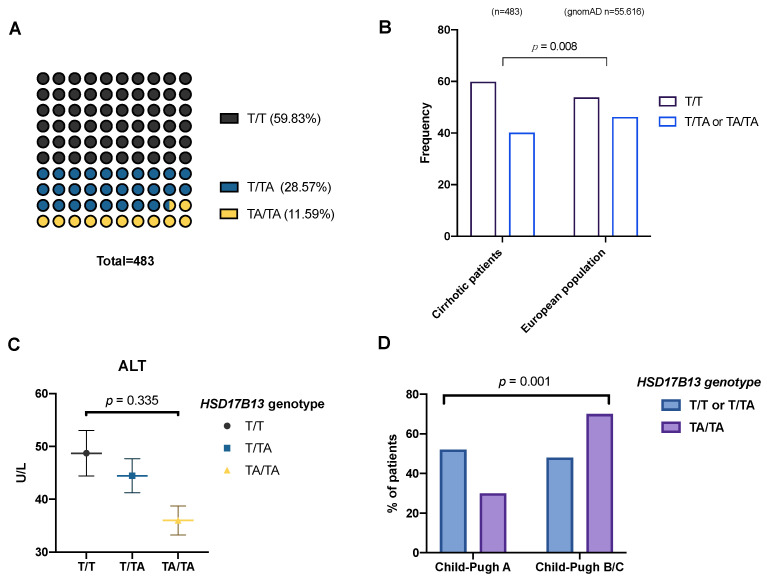
(**A**) Genotype frequency for rs72613567. (**B**) Comparison of minor allele frequency (MAF) across studies and in regards to liver function. (**C**) Alanine aminotransferase levels in patients with different genotypes (ANOVA). (**D**) Distribution of genotypes according to liver function measured by CTP score.

For the genotyping of rs72613567, we determined that 59.8% of patients were homozygous for the T allele, with 11.6% harboring the TA/TA variant (Figure 1A), resulting in a MAF (TA allele) of 0.255, comparable with that of previous studies (Abul-Husn et al., MAF = 0.242 [10]; Scheiner et al., MAF = 0.219) [14]. Nonetheless, we observed a deviation from the Hardy–Weinberg equilibrium (*p* < 0.05) for the variant in this cohort, which could indicate that the risk of disease *per se* is influenced by the polymorphism.

Compared to a control European population (publicly available from https://gnomAD.broadinsitute.org (accessed on 22 September 2022); *n* *=* 55,616), as in [13], the frequency of TA allele carriers was lower in cirrhotic patients (40.2% vs. 46.2%; *p* *=* 0.008), as shown in Figure 1B. This suggests, in agreement with previous reports, a protective effect of the variant against cirrhosis compared to healthy subjects. This effect was maintained for ALD (OR: 0.68; CI 95% 0.53–0.86; *p* *=* 0.001), but not for viral etiology (OR: 0.95; 95% CI, 0.72--1.25; *p* = 0.725). In addition, we observed the expected trend towards decreased ALT levels in patients with the TA allele (Figure 1C).

Curiously, the frequency of patients with worse liver function (CTP score B/C) was higher among those carrying the TA/TA genotype (Figure 1D). Furthermore, we also found that MELD scores (12.0 *±* 5.1 vs. 13.4 *±* 5.0; *p* *<* 0.05) and International Normalized Ratio (INR) levels (1.32 *±* 0.32 vs. 1.40 *±* 0.32; *p* *<* 0.05) were increased and albumin levels were decreased (35.4 *±* 7.8 vs. 32.3 *±* 7.6; *p* *<* 0.05) in rs72613567 TA/TA patients (Table 1).

### 2.2. Impact of rs72613567 Genotype on Mortality and Hepatic Decompensation

**Figure 2:**
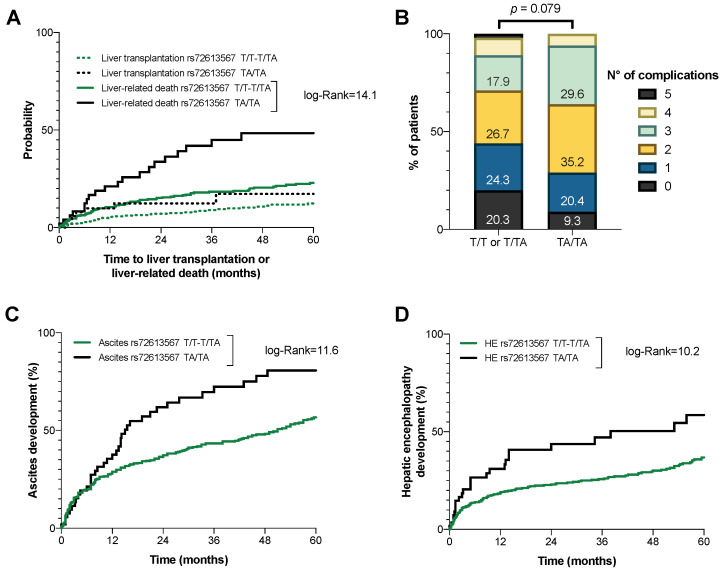
(**A**) Mortality with liver transplantation as a competing event. (**B**) Frequency of patients with an increasing number of cirrhosis complications. Cumulative incidence of (**C**) ascites and (**D**) hepatic encephalopathy development, according to the *HSD17B13* rs72613567 genotype.

The patients were followed for a median of 36.8 *±* 23.9 months. During that period, 47 patients (9.7%) underwent liver transplantation. Of the remaining patients, 111 (25.5%) died within five years, 92 of which (82.3% of the deaths) were related to their underlying liver disease. Surprisingly, carriers of the TA/TA genotype not only were not protected, but indeed had an increased risk of liver transplantation [HR 1.56 (95% CI 0.70–3.50); *p* *=* 0.275] and death [1.96 (1.22–3.15); *p* *=* 0.005], especially if taking into account liver-related deaths [2.52 (1.53–4.14); *p* *=* 0.001]. The cumulative incidence of liver-related death or transplantation is shown in Figure 2A. The resulting regression models, after adjusting for age, sex, liver function (MELD score), and factors influencing the progression of cirrhosis (i.e., alcohol intake or active viral infection), confirmed this trend (Table 2).

In terms of decompensation, patients with the variant in *HSD17B13* had an increased cumulative number of complications of cirrhosis (Figure 2B). Specifically, these patients had a higher risk of developing ascites (log-Rank 11.6; *p* *<* 0.001) and hepatic encephalopathy (log-Rank 10.2; *p* *<* 0.001) (Figure 2C,D). The Kaplan–Meier curves for the cumulative incidence of other complications, such as SBP (*p* *=* 0.059), EVB (*p* *=* 0.427), or hepatorenal syndrome (*p* *=* 0.236), did not differ significantly, probably due to the reduced number of cases in the cohort. Furthermore, and in contrast to recent studies, carriers of the TA/TA genotype were not protected from the 5-year development of HCC (log-Rank 0.1; *p* *=* 0.801).

### 2.3. Effect of Etiology and Interactions with PNPLA3 Genotype

Previous studies have adjudged HSD17B13 to have a role in lipid droplet metabolism, and, so far, most of the literature shows associations with non-viral liver disease (ALD/NAFLD patients). We wondered if the underlying etiology of cirrhosis could differentially affect the prognosis of patients harboring the variant rs72613567. In order to find out, we compared patients with ALD/NAFLD to those with viral etiology. Of those with steatohepatitis, only 8.2% (21/256) had NAFLD; viral etiology was comprised 148 (72.9%) patients with hepatitis C virus (HCV) and 55 (27.1%) with hepatitis B virus (HBV). As shown in Table 3, patients with viral hepatitis carrying the variant had worse liver function, measured by either Child–Turcotte–Pugh (CTP) (7.8 *±* 1.7 vs. 6.9 *±* 2.0; *p* *=* 0.038) or MELD (13.4 *±* 4.3 vs. 11.4 *±* 4.8; *p* *=* 0.065), at baseline than those with the major allele (T-). In addition, INR levels (*p* *=* 0.009) were higher, and albumin levels were decreased (*p* *=* 0.001). Although the trend was similar, there were no significant changes in baseline clinical and biochemical parameters among ALD/NAFLD patients, suggesting that the genotype may influence differently depending on the etiology.

The etiology seemed to influence the cumulative incidence of cirrhosis complications. Patients with viral hepatitis carrying the mutation had an increased risk of developing any complication at five years (87.0% vs. 57.4%; log-R 9.8; *p* *=* 0.002) than those with the major allele, but we did not observe a significant difference among patients with ALD/NAFLD, neither at one year (38.1% vs. 48.4%; *p* *=* 0.249) nor at five (64.5% vs. 57.0%; *p* *=* 0.363) years. However, carriers of the variant had reduced 5-year transplant-free survival, independently of the etiology (63.4% vs. 80.7%; log-R 5.3; *p* *=* 0.021 for ALD/NAFLD patients) (38.2% vs. 74.7%; log-R 9.0; *p* *=* 0.003 for viral hepatitis).

**Figure 3:**
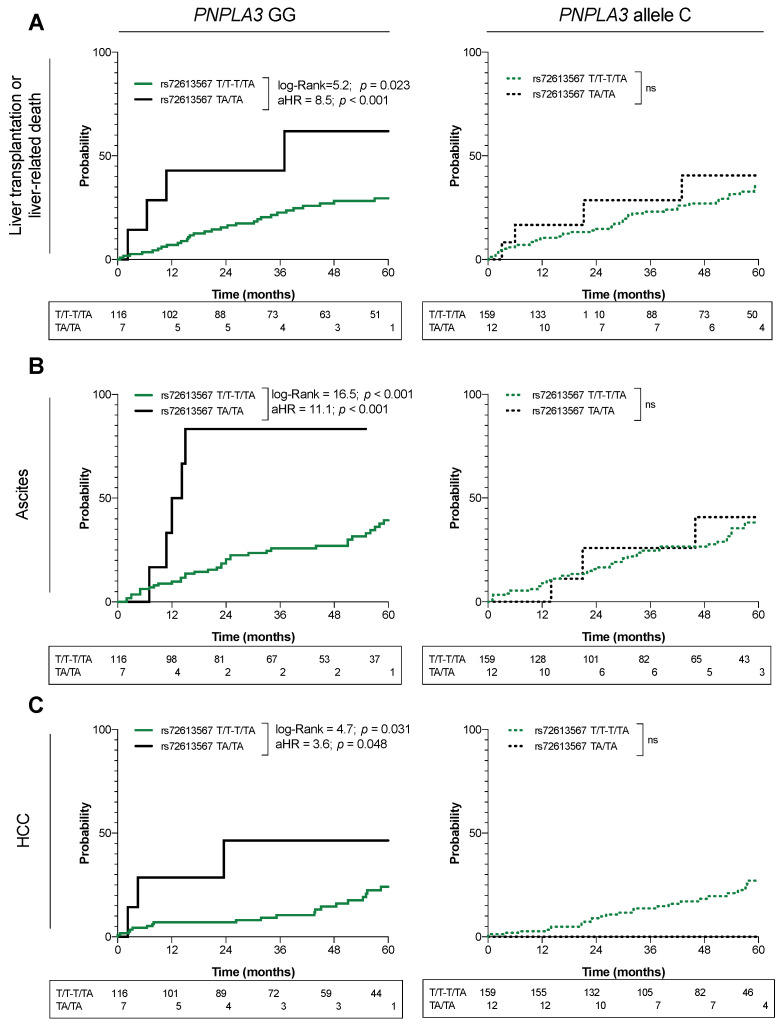
Effect of the *PNPLA3* rs738409 GG genotype on the risk of (**A**) liver-related mortality, (**B**) ascites, and (**C**) hepatocellular carcinoma, according to the *HSD17B13* rs72613567 genotype. aHR: adjusted hazard ratio; ns: not significant *p*-value.

Alternatively, we analyzed the interactions with the variant rs738409 (*PNPLA3*) in a subcohort of 294 patients. This polymorphism was in agreement with the Hardy–Weinberg equilibrium (*p* > 0.05). The result showed that the negative effect of the rs72613567-TA variant was present among carriers of the rs738409-GG genotype, who had a greater risk of liver-related mortality [aHR:8.550 (95% CI 1.490–21.864); *p* *<* 0.001], ascites [aHR:11.048 (3.83–32.129); *p* *<* 0.001], and HCC [aHR:3.641 (1.019–13.160); *p* *=* 0.048]. By contrast, this effect was diluted in non-carriers of the rs738409-GG genotype, and none of the outcomes were found to be significantly changed, although the rs72613567-TA/TA was still not protective. The cumulative incidences of these events are shown in Figure 3A–C.

## 3. Discussion

Several studies have revealed an association of epidemiological factors (geographic location, race, and environment), underlying different genetic backgrounds, with the prevalence of chronic liver diseases. Some of these genetic factors correlate with disease severity, progression to HCC, and liver-related outcomes. Usually, these mutations lead to structural, functional, or expression changes in the protein they code for, influencing a metabolic pathway that is differentially activated in disease progression. However, the liver, the most metabolically active and ductile organ, can go through different metabolic stages throughout liver disease progression, for the maintenance of hepatic regeneration and homeostasis [15]. Moreover, the physiopathology usually expands beyond the liver, and the consequences of these variants in peripheric tissues may also account for some of the risks, although they have not been properly evaluated yet.

The *HSD17B13* gene encodes for 17-beta-hydroxysteroid dehydrogenase 13, a retinol dehydrogenase highly expressed in the liver. Although its function is not completely clear, it is associated with lipid droplets, and it appears to be involved in the metabolism of lipids, as well as of steroid hormones, prostaglandins, xenobiotics, and retinoids [16]. The PNPLA3 protein also localizes to lipid droplets’ surfaces and plays a role in retinol metabolism; the I148M variant leads to increased fat accumulation in hepatic stellate cells, the main cellular site of retinol in the body. Retinol contributes to the regulation of gene transcription by binding nuclear hormone receptors (RAR/RXR), and the impairment induced by the variant in *PNPLA3* is associated with a proinflammatory and profibrogenic phenotype [17]. Contrariwise, although the variant in *HSD17B13* was linked with decreased levels of the spliced variant, it has been associated with protection against alcoholic and non-alcoholic fatty liver disease and against the progression towards fibrosis and HCC [18].

However, the findings of this work suggest that the variant rs72613567:TA in *HSD17B13* has no protective effect, but indeed increases the risk of decompensation and death in patients with advanced chronic liver disease. Previously, Ting et al. discussed the possibility of losing the protective effect given by the mutation once advanced fibrosis develops [19]; Scheiner et al. [14] showed, in agreement with our results, that the variant was not protective against hepatic decompensation and mortality among patients with portal hypertension. Numerous studies reported associations between genetics and hepatic decompensations [20,21]; nevertheless, for the first time, as far as we are concerned, we report here that the effect of the variant might change according to the stage of the disease.

Of note, most studies have been conducted on patients with ALD/NAFLD. Our cohort included few NAFLD patients (4.3% of total), and 42% were viral hepatitis patients, a prevalence that is consistent with epidemiological studies from the same period as this study [22], in which consecutive patients from outpatient clinics were included. In agreement with other studies, we showed that the underlying etiology of cirrhosis could impact the effect of the mutation on the prognosis, with a higher risk in patients with viral hepatitis. Certain differences in the pathophysiology could possibly explain this, such as the HCV lifecycle being closely linked to liver lipid metabolism [23]. In HBV patients, the mutation in *PNPLA3* has been associated with treatment response [24]. Further studies examining the interactions between 17-beta-HSD13 and the steps of HBV and HCV infection are required. In any case, the variant in *HSD17B13* did not show significant protection against liver-related outcomes in ALD/NAFLD patients.

Interestingly, the negative impact of the *HSD17B13* T>TA variant was diluted among non-carriers of the GG genotype of rs738409, suggesting an interaction between both polymorphisms, as previously reported [25,26]. Previous data showed a reduced effect of these variants on circulating retinol levels in patients with advanced chronic liver disease, in comparison to earlier stages [14]. Another study also observed that the impact of the variant in *PNPLA3* was different depending on the stage of the disease and the etiology [8]. These findings highlight the role of lipid droplets and retinol metabolites, as well as the fact that the metabolic plasticity of the liver is needed to maintain homeostasis through different stages of liver disease, and lead us to hypothesize that genetic variants disrupting these pathways may have a singular impact in the context of end-stage liver disease. In this context, the recent literature revealed some of the metabolic abnormalities taking place in liver cells of compensated and decompensated cirrhotic patients, such as impaired oxidative phosphorylation and fatty acid hypometabolism due to the inhibition of beta-oxidation [27].

Genetics is now considered a promising approach for the development of precision medicine, with great clinical applications for improving liver disease risk stratification, especially in NAFLD/ALD patients. There is also interest in exploring the pharmaceutical inhibition of 17-beta-HSD13 in order to diminish its steatogenic effect and prevent liver damage, just as the rs72613567:TA variant does. However, future studies analyzing the mechanistic effect of the genotype of *HSD17B13* (and other variants of interest) in different stages of the disease are warranted before being considered therapeutic targets with wide clinical applications.

This study has some limitations, mainly due to its retrospective nature. Nevertheless, we included a well-characterized, homogeneous cohort of cirrhotic patients that allowed us to assess the effect of the mutations in a specific setting. The majority of studies so far were population-based and limited in adjusting the results for liver function. Therefore, our sample size was modest compared to other studies, although a fair number of cases were included for some specific endpoints. Moreover, we only included individuals from our country; thus, the findings may not necessarily be extrapolated to other ethnicities, although the allele frequency observed here was similar to other studies using broader populations. Nevertheless, we acknowledge a deviation in HWE for *HSD17B13*; despite the possibility that it could be linked to methodological errors or non-random mating or sampling, this was taken into account, and it could indeed reflect an association with the disease.

In conclusion, the results of this study suggest that the protective effect conferred by the carriage of the *HSD17B13* rs72613567:TA genotype for liver disease may change in the context of cirrhosis, where it increased the risk of decompensation and death during the follow-up. A deeper understanding of the metabolic changes throughout the stages of advanced liver disease, focusing on the underlying effects of the *HSD17B13* genotype, is indispensable.

## 4. Materials and Methods

### 4.1. Experimental Design

Study population: A total of 483 patients suffering from liver cirrhosis of different etiologies were enrolled from 2007–2016 at the outpatient clinics of four different hospitals: Virgen del Rocio University Hospital, Valme University Hospital (Seville, Spain), Santa Creu i Sant Pau Hospital (Barcelona, Spain), and General University Hospital of Alicante (Alicante, Spain). The exclusion criteria were as follows: age *<* 18 years; absence of signed informed consent; active alcohol consumption; previous liver transplant or transjugular intrahepatic portosystemic shunt procedure; hepatocellular carcinoma diagnosed within the first 6 months of follow-up; and the presence of comorbidities with an estimated survival of less than 6 months at the time of enrollment. Cirrhosis was diagnosed according to the clinical practice guidelines [28].

The study was performed by following ethical guidelines expressed in the Declaration of Helsinki, and in the E18 (a guideline on genomic sampling and the management of genomic data) and E6 (a guideline for good clinical practice) from the International Council for Harmonization of Technical Requirements for Pharmaceuticals for Human Use. Demographic, clinical, and epidemiological data were recorded at the first visit, and all data were coded in the original clinical database. Human blood samples were collected after obtaining a signed informed consent form, as approved by the ethical committee of each hospital.

Definition of hepatic decompensation and transplant-free survival: Patients’ medical records were reviewed for the following events that defined (further) hepatic decompensation at baseline and during follow-up: large-volume paracentesis, spontaneous bacterial peritonitis (SBP), esophageal variceal bleeding (EVB), overt HE (West Haven grade > 1), hepatorenal syndrome, and liver-related death. Any (further) hepatic decompensation was defined by having at least one of these events occurring during follow-up. HCC was diagnosed without biopsy in the majority of the cases, because current clinical diagnostic approaches, including ultrasonography, computed tomography, or magnetic resonance imaging, were sufficient to diagnose HCC.

The patients were followed-up for up to five years, being censored on the day of death, or the day of the surgery if they were transplanted, for the calculation of transplant-free survival. Liver- and non-liver-related death and liver transplants were considered competing events for hepatic decompensation and HCC development.

### 4.2. DNA Genotyping

Genomic DNA was extracted from each sample of peripheral blood by using a MagNA Pure Compact Nucleic Acid Isolation Kit I. The concentration and purity of the DNA were evaluated by NanoDrop (ThermoFisher, Waltham, MA, USA). The genotyping of the single-nucleotide polymorphisms (SNP) of interest, *PNPLA3* rs738409 (assay ID: C_7241_10) and *HSD17B13* rs72613567 (custom TaqMan assay design), was performed using TaqMan SNP genotyping assays and chemistries (ThermoFisher, USA). PCRs were carried out, following the amplification protocol of FastStart Essential DNA Probes Master (Roche, Basel, Switzerland), in a QuantStudio 1 Real-Time PCR (ThermoFisher, USA). Genotype distribution was assessed for Hardy–Weinberg equilibrium by using a goodness-of-fit chi-square test; *p* > 0.05 was indicative of agreement with the equilibrium.

### 4.3. Statistical Analysis

Categorical variables are presented as percentages, while continuous variables are presented as means *±* standard deviation. The survival analysis was assessed by means of Cox regression for continuous variables, and by the Kaplan–Meier method, comparing subgroups by using the Mantel–Cox statistic, for categorical data. Adjusted multivariate analyses for the different outcomes were assessed by Cox regression, including age, sex, and variables with a known impact on prognosis, such as liver function measured by MELD score and etiology-dependent factors. These factors included the achievement of a sustained virological response for viral hepatitis, or the moderate relapse in alcohol intake for alcoholic hepatitis (any severe relapse leading to hospitalization was retrospectively excluded from the analysis). The strength of association between the variables and the outcomes under each model was represented by hazard ratios with 95% CIs. Missing data that were considered as missing completely at random biologically implausible values were set to missing values. Variables with <5% of missing data were imputed by multiple lineal regression, and variables with >5% of missing data were dropped. A two-sided *p*-value of <0.05 was set as the threshold for statistical significance. SPSS (v.25.0; SPSS, Inc., Chicago, IL, USA) and STATA (v.16, StataCorp LLC, College Station, TX, USA) statistical packages were used for analyses, and graphics were performed by using a GraphPad PRISM (v.8.0, GraphPad Software, Inc., San Diego, CA, USA).

## Figures and Tables

**Table 1:** Comparison of clinical and demographic characteristics of the patients according to the genotype of *HSD17B13* rs72613567.

Variable	Total (*n* = 483)	HSD17B13 T/T or T/TA (*n* = 427)	TA/TA (*n* = 56)	*p*-Value
Age ± SD, years	59.4 ± 11.9	59.2 ± 11.5	60.8 ± 14.8	0.448
Male sex	68.7% (332/483)	68.1% (291/427)	73.2% (41/56)	0.442
CTP	7.0 ± 1.9	6.9 ± 1.9	7.6 ± 1.8	0.014
Etiology				0.800
Alcohol	48.7% (235/483)	49.2% (210/427)	44.6% (25/56)	
Viral	42.0% (203/483)	41.7% (178/427)	44.6% (25/56)	
Other	9.3% (45/483)	9.1% (39/427)	10.7% (6/56)	
MELD ± SD	12.2 ± 5.1	12.0 ± 5.1	13.4 ± 5.0	0.047
Albumin ± SD (g/L)	35.0 ± 7.9	35.4 ± 7.8	32.2 ± 7.6	0.004
Bilirubin ± SD (mg/dL)	2.14 ± 2.17	2.13 ± 2.14	2.24 ± 2.46	0.727
Platelets ± SD (×10^9^/L)	121.3 ± 70.0	120.8 ± 64.9	133.1 ± 100.9	0.340
Creatinine ± SD (mg/dL)	0.98 ± 0.45	0.98 ± 0.46	0.96 ± 0.36	0.762
AST ± SD (IU/L)	60.5 ± 57.8	61.5 ± 60.4	53.3 ± 31.9	0.315
ALT ± SD (IU/L)	46.0 ± 60.5	47.3 ± 63.9	36.0 ± 20.6	0.188
INR ± SD	1.32 ± 0.32	1.32 ± 0.32	1.40 ± 0.32	0.046
Leukocytes ± SD (cells/×10^9^/L)	6.96 ± 5.99	7.00 ± 6.19	6.63 ± 4.12	0.681

**Table 2:** Multivariate Cox regression analyses on the impact of the genotype of *HSD17B13* rs72613567 on liver transplantation, mortality, and hepatic decompensation.

	Liver Transplantation		Mortality		Liver-Related Death		Any (Further) Decompensation	
	aHR (95% CI)	*p*-Value	aHR (95% CI)	*p*-Value	aHR (95% CI)	*p*-Value	aHR (95% CI)	*p*-Value
Sex	1.96 (0.87–4.32)	0.097	1.17 (0.71–1.93)	0.531	1.27 (0.72–2.21)	0.404	1.04 (0.64–1.70)	0.880
Age	0.98 (0.95–1.00)	0.089	1.05 (1.03–1.07)	0.001	1.05 (1.03–1.08)	0.001	1.04 (1.02–1.06)	0.001
MELD	1.09 (1.04–1.15)	0.001	1.09 (1.05–1.14)	0.001	1.11 (1.07–1.16)	0.001	1.29 (1.21–1.38)	0.001
Etiological factors	1.52 (0.81–2.84)	0.197	1.52 (0.97–2.39)	0.069	1.39 (0.84–2.30)	0.204	1.99 (1.29–3.07)	0.002
rs72613567 TA/TA genotype	1.21 (0.49–2.93)	0.673	1.67 (0.91–3.18)	0.089	2.32 (1.20–4.46)	0.012	2.37 (1.09–5.06)	0.029

MELD: Model for End-Stage Liver Disease; etiological factors: relapse in alcohol intake or active viral infection.

**Table 3:** Comparison of patients’ characteristics according to the genotype of *HSD17B13*, depending on the etiology of the cirrhosis.

Variable	ALD/NAFLD (*n* = 256)			Viral Hepatitis (*n* = 203)		
	HSD17B13		*p*-Value	HSD17B13		*p*-Value
	T/T or T/TA	TA/TA		T/T or T/TA	TA/TA	
Age ± SD, years	59.1 ± 10.1	58.8 ± 13.2	0.853	59.3 ± 13.2	63.2 ± 16.4	0.259
Male sex	76.9% (176/229)	81.4% (22/27)	0.316	59.0% (105/178)	64.0% (16/25)	0.442
CTP	6.8 ± 1.9	7.4 ± 1.9	0.167	6.9 ± 2.0	7.8 ± 1.7	0.038
MELD ± SD	12.3 ± 5.2	13.8 ± 5.6	0.236	11.4 ± 4.8	13.2 ± 4.3	0.065
Albumin ± SD (g/L)	35.8 ± 7.7	34.1 ± 8.4	0.309	35.1 ± 8.1	29.7 ± 6.2	0.001
Bilirubin ± SD (mg/dL)	2.2 ± 2.37	2.18 ± 1.90	0.816	1.94 ± 1.73	2.33 ± 3.05	0.346
Platelets ± SD (×10^9^/L)	125.3 ± 65.4	141.1 ± 69.1	0.279	112.1 ± 63.5	125.9 ± 131.5	0.610
Creatinine ± SD (mg/dL)	0.98 ± 0.49	0.98 ± 0.42	0.739	0.95 ± 0.40	0.95 ± 0.25	0.938
AST ± SD (IU/L)	54.7 ± 63.9	48.5 ± 29.4	0.620	71.3 ± 56.3	59.5 ± 34.5	0.121
ALT ± SD (IU/L)	36.1 ± 42.5	31.8 ± 19.1	0.585	63.3 ± 83.5	41.2 ± 21.9	0.191
INR ± SD	1.34 ± 0.34	1.39 ± 0.31	0.555	1.27 ± 0.27	1.44 ± 0.32	0.009
Leukocytes ± SD (cells/×10^9^/L)	7.11 ± 4.58	8.19 ± 4.87	0.328	6.93 ± 8.18	5.10 ± 2.64	0.293

## Data Availability

The data presented in this study are available upon request from the corresponding author. The data are not publicly available due to privacy reasons.
